# Occurrence, load and transport-associated risk factors of *Salmonella* spp. in chickens sold in informal live bird markets in Centre-Nord Region, Burkina Faso

**DOI:** 10.1016/j.psj.2026.107886

**Published:** 2026-10-05

**Authors:** Wendenso Patrick Bertrand Tiendrébéogo, Assèta Kagambèga, Arshnee Moodley, Tho Abou Da, Rasmané Tao, Brice Ouedraogo, Nicolas Barro, Michel Dione

**Affiliations:** a Laboratory of Molecular Biology, Epidemiology and Surveillance of Foodborne Bacteria and Viruses, Department of Biochemistry-Microbiology, Doctoral School of Science and Technology, Joseph KI-ZERBO University, Ouagadougou, 03 BP 7021, Burkina Faso; b École Normale Supérieure, Ouagadougou, Burkina Faso; c International Livestock Research Institute, HEALTH program, Nairobi, Kenya; d University of Copenhagen, Department of Veterinary and Animal Science, Frederiksberg C, Denmark; e International Livestock Research Institute, HEALTH program, Ouagadougou, Burkina Faso

**Keywords:** Salmonella spp., Poultry collectors, Live-bird markets, Food safety, Biosecurity

## Abstract

Informal poultry trade is central to live-bird value chains in sub-Saharan Africa, but transport and handling practices may facilitate the spread of zoonotic pathogens such as *Salmonella* spp. This regional study assessed the occurrence, bacterial load, and transport-related risk factors of *Salmonella* spp. in indigenous village chickens (*Gallus gallus domesticus*) marketed through the Kaya live-bird market in the Centre-Nord Region of Burkina Faso.

A cross-sectional survey was conducted among 49 poultry collectors. Four clinically healthy indigenous chickens (*Gallus gallus domesticus*) were selected from each collector’s transported batch using a stratified random sampling procedure based on spatial position (front, middle, rear) yielding a total of 196 birds. Birds originated from smallholder households in surrounding villages and were selected for live sale. Cecal contents were analyzed for *Salmonella* spp. using ISO 6579:2012 protocols, bacterial loads were quantified by the Most Probable Number method, and isolates were confirmed by MALDI-TOF MS. Structured interviews documented collector characteristics, sourcing, transport, hygiene, and biosecurity. Logistic regression identified factors associated with *Salmonella* occurrence, while linear regression assessed predictors of bacterial load.

*Salmonella* spp. was detected in 41.3% of chickens, with 59.2% of collectors having at least one positive bird. Transporting 21-50 birds and collectors having verified the vaccination status of birds when collecting chickens were associated with higher odds of detection. Higher bacterial loads were linked to temporary stops during collection or transport and reuse of dedicated work clothing without proper cleaning.

This regional survey highlights the collector and live-bird market stage as a critical interface for *Salmonella* circulation in village poultry systems. Practical recommendation include reducing overcrowding during transport, improving confinement and hygiene practices, and strengthening biosecurity measures among poultry collectors.

## Introduction

Village poultry production is an important component of household livelihoods in Burkina Faso, where chickens are widely raised in traditional extensive systems and contribute to food supply, cash income, and day-to-day resilience (Ayssiwede et al., 2013; FAO, 2018). In the Centre-Nord region, these birds move through largely informal trading networks linking rural villages to urban consumers. Kaya, which hosts the largest live bird market in the region, is a major hub in this system because it receives chickens collected from multiple surrounding villages and redistributed to traders and households.

This movement of live birds has important food safety and public health implications. Bird collectors purchase and transport chickens from different household flocks, often under conditions that involve crowding, repeated handling, prolonged confinement, limited cleaning of transport materials, and mixing of birds from multiple origins. These practices may facilitate faecal contamination, cross-contamination between birds, and exposure of traders, handlers, consumers, and the market environment to enteric pathogens, including *Salmonella* spp. (Coker et al., 2002; Kagambèga et al., 2018; Ofori et al., 2023).

*Salmonella* spp. is a major cause of foodborne disease globally, and poultry is a recognized reservoir (Majowicz et al., 2010; Dallman et al., 2016). In Burkina Faso, previous studies have documented *Salmonella* spp. contamination in poultry and poultry environments, but most available evidence focuses on household, farm, or carcass-level contamination (Tiendrébéogo et al., 2026). Less is known about the collector stage of the value chain, despite the fact that collectors may act as a bridge between household production systems and urban live-bird markets. The collector stage is epidemiologically important for several reasons. First, collectors aggregate birds from multiple households with different hygiene, management, and disease histories. Second, transport and handling conditions may increase stress, faecal shedding, and opportunities for cross-contamination. Third, live birds are handled by multiple actors before sale, slaughter, or household preparation. Understanding risks at this interface is therefore essential for designing feasible, value-chain-based interventions (Grace, 2015; Thomas et al., 2020; Ramtahal et al., 2022).

Another important gap concerns quantification. Most studies report *Salmonella* spp. prevalence, but bacterial load may provide additional insight into contamination intensity and potential transmission risk (Van Immerseel et al., 2005; Foley et al., 2011; Berghaus et al., 2013). This study aimed to estimate the occurrence and bacterial load of *Salmonella* spp. in chickens handled by live-bird collectors in Kaya, Burkina Faso, and to identify collector- and transport-related factors associated with *Salmonella* spp. detection and bacterial load. We hypothesized that bird aggregation, transport density, temporary confinement, and hygiene-related practices would be associated with *Salmonella* spp. contamination among chickens handled by collectors.

## Materials and methods

### Study area

The study was conducted in Kaya, located in the Province of Sandbondtenga, in the Centre-Nord region of Burkina Faso (currently Kuilsé region). The commune covers approximately 922 km² and comprises seven urban sectors and 71 attached villages. According to the 2019 General Population and Housing Census (RGPH), the population of Kaya was 207,740 inhabitants distributed across 40,243 households, of whom 99,016 were men (47.67%) and 108,724 were women (52.33%) (Fig. 1). Kaya is an important urban and commercial center in north-central Burkina Faso and serves as the provincial capital of Sandbondtenga (MHU, 2020).

**Fig. 1:**
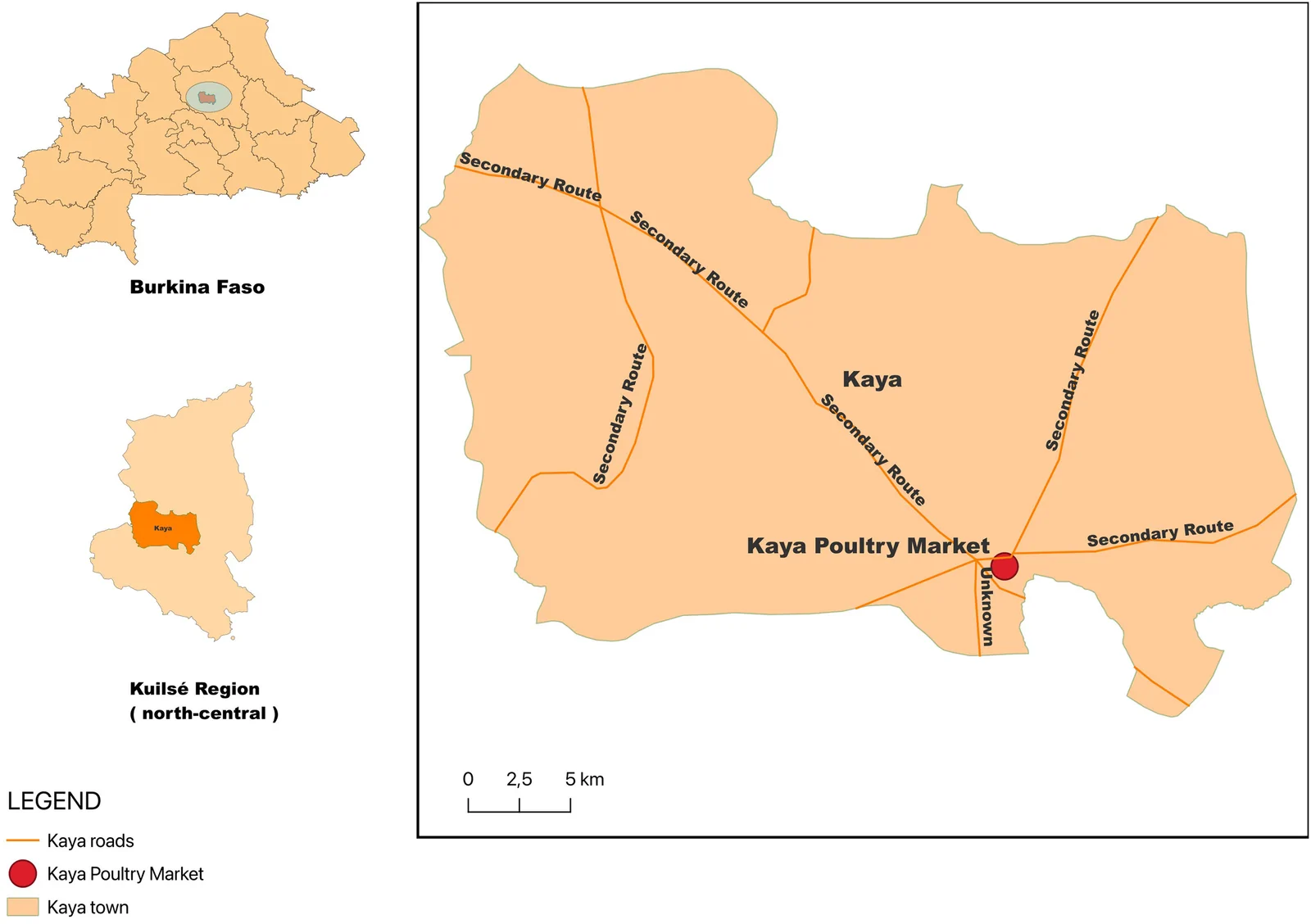
Map of the study area.

Kaya hosts the largest live poultry market in the region and serves as the main destination for poultry collectors returning from surrounding villages after collection activities. As such, the market functions as a major collection, transit, and redistribution point where indigenous chickens raised under traditional extensive village production systems are assembled from multiple rural sources, handled, transported, and sold under largely informal conditions.

### Sampling strategy and participant recruitment

Access to live-bird collectors was facilitated through collaboration with the president of the poultry traders’ association at the Kaya live bird market, which is the principal poultry market in the Centre-Nord region. The research team positioned itself at the market and waited for collectors upon their return from poultry collection activities in surrounding villages, between 9:00 and 11:00 a.m. Collectors were recruited consecutively on major market days, which occur every three days, between 11 April and 23 May 2024, during the dry season. Collectors who met the inclusion criteria were invited to participate. Participation was voluntary, and all eligible collectors present during the study period were invited to take part. A total of 49 collectors were approached, and all agreed to participate, resulting in a participation rate of 100%. The study targeted clinically healthy indigenous chickens (*Gallus gallus domesticus*) originating from villages surrounding Kaya, and raised under traditional extensive production systems. These birds were collected by the participating traders and transported to the Kaya live bird market for sale. Thus, the sampling unit was the individual chicken, while the collector represented the clustering unit for the epidemiological analysis.

### Sample size calculation

To assess impacts of transport and market practices on pathogen shedding (*Salmonella* spp..), morbidity, mortality and welfare, 196 chickens were sampled from 49 traders at end-markets. Sample size was calculated as follows: If prevalence of free range chicken post transport at the market = 0.52 (McCrea et al., 2006) with a precision of 7% and a 95% confidence level, a minimum sample size of 196 chickens was estimated. Accordingly, 196 chickens were sampled from 49 collectors, with four chickens sampled per collector. The sampling design therefore allowed assessment of individual level *salmonella* detection while accounting for the clustering of chickens within collectors during the statistical analysis.

### Selection and collection of chicken feces

Data collection and biological sampling were conducted during a single visit with each participating collector. A structured questionnaire was first administered to collection socio-demographic characteristics of collectors, poultry trading practices, transport conditions, hygiene measures, and biosecurity behaviors. Immediately after the interview, biological sampling was performed. From each collector’s transported batch, four clinically healthy indigenous village chickens (*Gallus gallus domesticus*) were selected using a stratified random sampling approach based on the spatial position of chickens within the batch. Each batch was divided into three zones (front, middle, rear). One bird was randomly chosen from the front zone, two from the middle zone, and one from the rear zone. This procedure was consistently applied to all participating collectors, resulting in four chickens per collector and a total of 196 birds sampled.

Because birds originated from multiple villages and were aggregated by collectors, individual-level information (e.g., age, treatment history, or vaccination status at purchase) was not available for each bird. Vaccination status recorded in the questionnaire referred to the collectors’ practice of considering vaccination status when purchasing birds and concerned Newcastle disease, the vaccination most commonly practiced in the study setting. Selected chickens were purchased and humanely euthanized on site by cervical dislocation by trained personnel. Cecal contents were aseptically collected using sterile gloves, placed into labeled sterile bags, and stored in insulated coolers at 4 °C and transported to the Laboratory of Molecular Biology, Epidemiology, and Surveillance of Foodborne Bacteria and Viruses (LaBESTA) at Joseph Ki-Zerbo University for processing within 4 h.

### Microbiological analyses

#### Detection of Salmonella spp

Detection of *Salmonella* spp. was performed according to the ISO 6579:2017. One gram of cecal content was pre‑enriched in 9 mL of sterile Buffered Peptone Water (BPW) at 37 °C for 24 h. Selective enrichment was performed by transferring 100 µL of the pre-enrichment culture into 9.9 mL of Rappaport–Vassiliadis (RV) broth and incubated at 41.5 °C for 24 h which falls within the ISO 6579‑1:2017 recommended range of 41.5 ± 0.5 °C. Isolation was achieved by streaking 10 µL from each RV tube Enriched cultures (10 µL) were streaked onto *Salmonella* chromogenic agar plates (Condalab, Cat. No. 1122) and incubated at 37 °C for 24 h. Presumptive colonies, typically appearing as mauve to purple colonies on chromogenic agar, were confirned using biochemical tests: Kligler‑Hajna iron agar, Triple Sugar Iron, urease, and indole tests. All confirmed isolates were further identified using matrix‑assisted laser desorption/ionization time‑of‑flight mass spectrometry (MALDI-TOF MS) on the Bruker Biotyper system (Bruker Daltonik GmbH), using the direct spotting technique following the manufacturer’s instructions. Confirmed isolates were preserved at −80 °C in cryovials containing 15% glycerol.

#### Quantification *of Salmonella* spp

For *Salmonella* spp. load estimation, a modified Most Probable Number (MPN) method was used, adapted from Dunn et al. (2022). Briefly, 1 g of cecal content was homogenized in 9 mL of buffered peptone water (BPW) by vortex mixing. Serial ten-fold dilutions (10⁻² - 10⁻³) were prepared in BPW, three replicate tubes per dilution level and subjected to pre-enrichment at 37 °C for 18 h. From each pre-enrichment, 0.1 mL was transferred into 9.9 mL of Rappaport-Vassiliadis broth and incubated at 41.5 °C for 24 h. Following selective enrichment, aliquots were streaked onto *Salmonella* chromogenic agar (Condalab, Cat. No. 1122) and incubated at 37 °C for 24 h. Presumptive colonies were confirmed using standard biochemical assays as described above. The pattern of positive tubes across dilution levels was used to estimate bacterial concentrations, expressed as MPN/g, following the maximum-likelihood approach described by Jarvis et al. (2010) and Blodgett (2023).

### Data analysis

#### Prevalence estimation

Animal‑level prevalence was calculated as the proportion of positive samples among all sampled chickens. Collector‑level prevalence was calculated as the proportion of collectors with at least one positive sampled bird. Exact 95% confidence intervals (CI) were calculated using the Clopper–Pearson method.

#### Risk factor analysis

All analyses were performed using Stata version 14.0 (StataCorp, College Station, TX, USA), with RStudio (version 2025.05.1, Foundation for Statistical Computing, Vienna, Austria) software used for graphical visualization. Given the clustered design (four samples per collector), mixed-effects models with a random intercept for collector identity were applied to account for within collector correlation.

Two outcomes were analyzed: (i) *Salmonella* spp. detection (occurrence) was analysed as a binary outcome using mixed-effects logistic regression, with collector identity included as a random intercept to account for clustering of chickens within collectors. Its inclusion was supported by a likelihood ratio test (LR χ²(5) = 12.27, *p* = 0.031). Results were expressed as odds ratios (OR) and 95% CIs, and (ii) bacterial load was analysed using mixed-effects linear regression fitted on positive samples only. For descriptive interpretation of bacterial load, *Salmonella* spp. MPN were grouped into four categories: undetectable (0 MPN/g), low (>0 to ≤0.5 MPN/g), moderate (>0.5 to ≤3 MPN/g), and high (>3 MPN/g) contamination levels based on the distribution of observed values.

Variable selection involved univariable screening (*p* < 0.20) followed by backward elimination based on likelihood ratio tests and the Akaike Information Criterion. Continuous variables were assessed for linearity and categorized when appropriate based on their distribution and epidemiological relevance.

Model performance for the prevalence analysis was evaluated using the Hosmer–Lemeshow goodness-of-fit test and the area under the receiver operating characteristic curve (AUC). The degree of clustering was quantified using the intraclass correlation coefficient (ICC), and bootstrap resampling was used for internal validation. Sensitivity analyses compared mixed-effects results with standard regression models.

Statistical significance was set at *p* < 0.05 (two-sided), without correction for multiple testing given the exploratory nature of the study.

## Results

### Live bird market operation

All participating collectors transported chickens by tying birds by the legs and suspending them on both sides of motorcycles (Fig. 2). Other transport methods were also observed, including poultry baskets (40.8%), wooden crates (34.7%), and plastic crates (2.0%), often used in combination with tying birds directly to motorcycles.

**Fig. 2:**
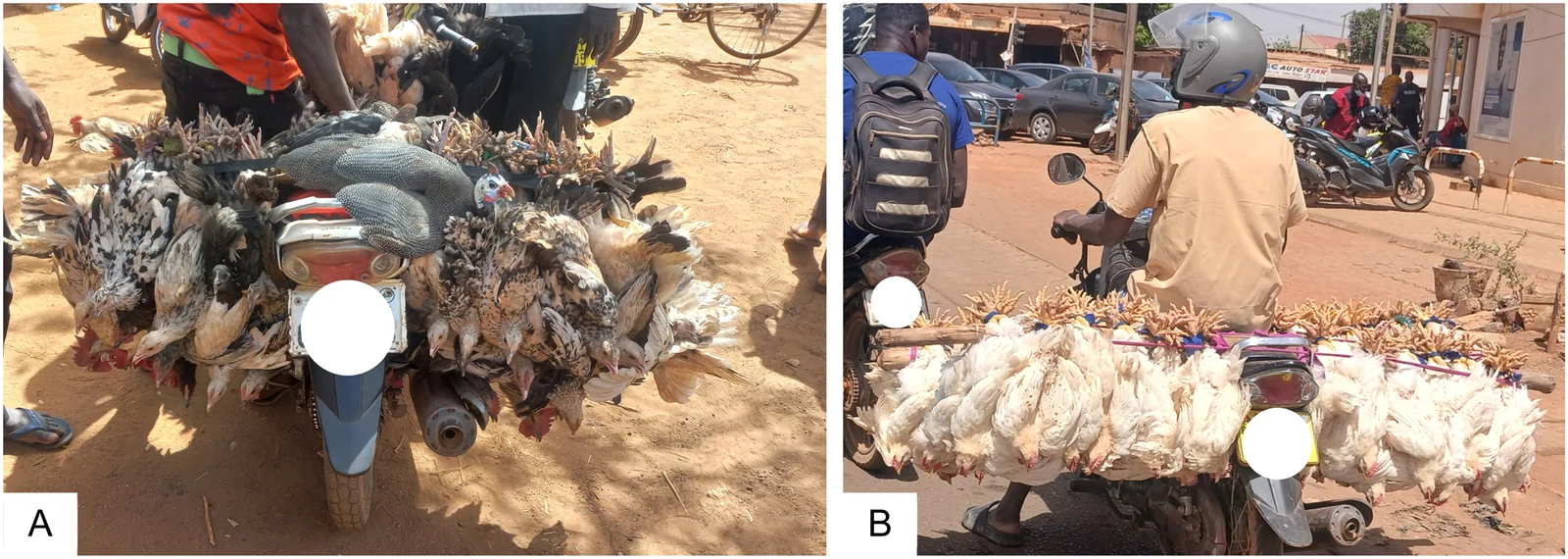
**Examples of poultry transport practices observed among collectors in Kaya, Burkina Faso. (A)** Transport of mixed poultry under overcrowded conditions on a motorcycle during collection activities. **(B)** Transport of chickens tied by the legs and carried on a motorcycle in an urban setting.

Transport conditions frequently involved crowding, close physical contact between birds, and mixing of birds from different sources.

At the market level, birds were rapidly transferred from collectors to traders. Upon arrival, collectors were often surrounded by traders who selected birds directly from the transported batches. In some cases, birds were unloaded onto the ground before selection. Birds from different sources were often kept in close proximity, either on the ground or in cages, with repeated handling by multiple individuals. Fig. 2 illustrates transport practices observed among collectors, with chickens from different sources carried together on motorcycles, often tied by the legs and in overcrowded conditions.

### Live bird collectors sociodemographic characteristics

Table 1 presents the sociodemographic characteristics of the 49 collectors included in the study. The age distribution was relatively balanced, with the largest proportions observed among collectors aged 30–39 years (28.6%) and 40–49 years (28.6%), followed by those under 30 years (20.4%) and those aged ≥50 years (22.4%). Most collectors were married (81.6%), while a smaller proportion were single (18.4%). In terms of education, nearly half of the collectors had no formal education (44.9%), and the remainder had primary education (26.5%), informal literacy (22.4%), or secondary education (6.1%), indicating generally low levels of formal schooling in this population. Poultry trade was the main source of income for the vast majority of collectors (83.7%), with only a few engaged primarily in breeding (8.2%) or agriculture (8.2%). Regarding experience, a substantial proportion of collectors reported long-term involvement in trading, with 40.8% having ≥20 years of experience, while 28.6% had 10–19 years and 30.6% had less than 10 years of experience.

**Table 1:** Sociodemographic profile of bird collectors (*n* = 49).

Variable	Category	N (collectors)	%
**Gender**	Male	49	100.0
**Age**	<30 years	10	20.4
	30–39 years	14	28.6
	40–49 years	14	28.6
	≥50 years	11	22.4
**Marital status**	Married	40	81.6
	Single	9	18.4
**Education level**	No formal education	22	44.9
	Primary	13	26.5
	Literate (informal)	11	22.4
	Secondary	3	6.1
**Primary income source**	Poultry trade	41	83.7
	Other livestock breeding	4	8.2
	Crop farming	4	8.2
**Poultry trading experience**	<10 years	15	30.6
	10–19 years	14	28.6
	≥20 years	20	40.8

### *Salmonella* spp. prevalence and associated factors

#### Occurrence *Salmonell*a spp

Of the 196 chickens sampled, 81 were positive for *Salmonella* spp., corresponding to an animal‑level prevalence of 41.3% (95% CI: 34.4-48.5). At the collector level, 29 of the 49 collectors had at least one positive bird, corresponding to a collector‑level prevalence of 59.2% (95% CI: 45.2-72.1).

Among all samples, 115 (58.7%) had no detectable *Salmonella* spp. Of the total 196 samples, 58 (29.6%) had low loads (>0–0.5 MPN/g), 17 (8.7%) had moderate loads (0.5–3 MPN/g), and 6 (3.1%) high loads (>3 MPN/g).

### Risk factors associated with *Salmonella* spp. presence at the animal level

#### Univariable logistic regression

In the univariable analysis, several variables were associated with Salmonella spp. detection, including reported consideration of vaccination status, number of birds transported, distance-related variables, and cage-cleaning category (Table 2). Collectors who reported considering the vaccination status of chickens at purchase was significantly linked to higher odds of contamination (OR = 2.68, 95% CI: 1.11–6.46, *p* = 0.029). Transporting 21–50 birds was also associated with increased risk (OR = 4.03, 95% CI: 1.49–10.95, *p* = 0.006), compared to transporting 1–20 birds. In contrast, a minimum farm‑to‑market distance of 11–15 km was protective (OR = 0.23, 95% CI: 0.08–0.67, *p* = 0.007). Similarly, a furthest distance of 20–40 km reduced the odds of contamination (OR = 0.48, 95% CI: 0.23–0.97, *p* = 0.041). Other factors such as age, marital status, education level, trading experience, biosecurity training, and purchase of sick poultry showed no significant associations. Practices like resting during transport and general transport practices demonstrated borderline associations but did not reach statistical significance.

**Table 2:** Results of univariable analyses of factors associated with Salmonella spp. presence in chicken.

Variable	Reference category	Comparison category	OR	95% CI	p-value
**Age**	<30 years	<30 years	1.00	—	0.709
		30–49 years	0.88	[0.43 – 1.83]	0.739
		≥50 years	0.70	[0.29 – 1.68]	0.421
**Marital status**	Married	Married	1.00	—	0.427
		Single	1.34	[0.65 – 2.78]	0.427
**Education level**	Literate	Literate	1.00	—	0.514
		No formal education	1.69	[0.80 – 3.57]	0.173
		Primary	1.31	[0.57 – 3.02]	0.526
		Secondary	0.97	[0.25 – 3.74]	0.961
**Primary income source**	Agriculture	Agriculture	1.00	—	0.157
		Breeding	3.67	[0.85 – 15.84]	0.082
		Poultry trade	1.48	[0.49 – 4.46]	0.485
**Trading experience**	<10 years	<10 years	1.00	—	0.923
		10–19 years	0.91	[0.44 – 1.91]	0.805
		≥20 years	0.87	[0.44 – 1.72]	0.692
**General information**	No	No	1.00	—	0.248
		Yes	0.71	[0.40 – 1.26]	0.248
**Purchase time**	Morning (<10:00)	Morning (<10:00)	1.00	—	0.048
		Evening (>17:00)	4.52	[0.89 – 22.99]	0.069
**Vaccination status**	No	No	1.00	—	0.029*
		Yes	2.68	[1.11 – 6.46]	0.029*
**Buys sick poultry**	No	No	1.00	—	0.259
		Yes	0.71	[0.39 – 1.29]	0.259
**Transport practices**	No	No	1.00	—	0.070
		Yes	2.11	[0.94 – 4.75]	0.070
**Birds transported**	1–20	1–20	1.00	—	0.037
		21–50	4.03	[1.49 – 10.95]	0.006**
		51–80	1.77	[0.58 – 5.41]	0.320
		81–120	2.44	[0.81 – 7.36]	0.112
		>120	3.67	[0.42 – 31.73]	0.238
**Farm distance**	<15 km	<15 km	1.00	—	0.340
		15–25 km	0.68	[0.34 – 1.37]	0.282
		>25 km	1.13	[0.55 – 2.28]	0.744
**Furthest distance**	<20 km	<20 km	1.00	—	0.076
		20–40 km	0.48	[0.23 – 0.97]	0.041*
		>40 km	0.83	[0.36 – 1.92]	0.670
**Minimum distance**	0–5 km	0–5 km	1.00	—	0.044
		6–10 km	0.76	[0.33 – 1.76]	0.522
		11–15 km	0.23	[0.08 – 0.67]	0.007**
		16–25 km	0.68	[0.30 – 1.52]	0.346
		>25 km	1.18	[0.44 – 3.20]	0.743
**Resting during transport**	No	No	1.00	—	0.195
		Yes	1.85	[0.73 – 4.70]	0.195
**Cage cleaning**	Does not clean	Does not clean	1.00	—	0.059
		Whenever poultry is to be transported	2.42	[0.91 – 6.44]	0.075
		Monthly	5.78	[0.58 – 57.34]	0.134
		Weekly	1.93	[0.67 – 5.51]	0.221
**Biosecurity training**	No	No	1.00	—	0.430
		Yes	1.27	[0.70 – 2.33]	0.430

OR : Odds Ratio; CI : Confidence Interval.

Significance levels : * *p* < 0,05; ** *p* < 0,01; *** *p* < 0,001.

#### Multivariate logistic regression model

In the multivariate (crude) logistic regression, vaccination status and transporting 21–50 birds were significantly associated with higher odds of *Salmonella* spp. positivity (Table 3). Collectors who reported interest in the vaccination status of poultry had nearly three times higher odds of contamination compared to those who did not (OR = 2.81, 95% CI: 1.09–7.25, *p* = 0.032). Similarly, transporting 21–50 birds simultaneously increased the odds of detection by almost fourfold compared to transporting 1–20 birds (OR = 3.68, 95% CI: 1.35–10.05, *p* = 0.011). No significant differences were observed for larger flock sizes (51–80, 81–120, or >120 birds).

**Table 3:** Factors associated with *Salmonella* spp*.* prevalence: comparison of standard and mixed-effects logistic regression models (*N* = 196 samples).

Variable	Category	OR (crude)	95% CI	p-value	OR (mixed)	95% CI	p-value
**Vaccination status**	No (ref)	1.00	—	—	1.00	—	—
	Yes	2.81	1.09–7.25	0.032*	3.02	1.01–9.05	0.048*
**Birds transported**	1–20 (ref)	1.00	—	—	1.00	—	—
	21–50	3.68	1.35–10.05	0.011*	3.96	1.28–12.23	0.017*
	51–80	1.57	0.51–4.87	0.431	1.60	0.46–5.62	0.463
	81–120	1.76	0.56–5.60	0.335	1.82	0.50–6.55	0.361
	>120	3.67	0.42–31.7	0.238	3.92	0.34–45.4	0.274

OR: Adjusted Odds Ratio; CI: Confidence Interval.

Significance levels : * *p* < 0,05; ** *p* < 0,01; *** *p* < 0,001.

In the final mixed‑effects logistic regression model, which accounted for clustering of chickens within collectors, these associations remained significant. Vaccination status was associated with higher odds of *Salmonella* spp. detection (adjusted OR = 3.02, 95% CI: 1.01–9.05, *p* = 0.048), and transporting 21–50 birds was linked to approximately four times higher odds of positivity (adjusted OR = 3.96, 95% CI: 1.28–12.23, *p* = 0.017). Larger flock sizes did not show significant associations. Model diagnostics indicated adequate calibration (Hosmer–Lemeshow χ² = 5.62, df = 4, *p* = 0.229) and moderate discriminatory ability (AUC = 0.644). The intraclass correlation coefficient was 0.070 (95% CI: 0.005–0.532), suggesting limited but non‑negligible clustering at the collector level. Results from the standard logistic regression were consistent with those from the mixed‑effects model.

### Factors associated with *Salmonella* spp. bacterial load: linear regression analysis

In the final mixed‑effects linear regression model, temporary stops during collection or transport, during which birds remained confined in transport conditions, were strongly associated with higher log-transformed Salmonella spp. load. Commercial trading activity was associated with a borderline increase in bacterial load (β = 0.769, 95% CI: −0.007–1.546, *p* = 0.052). Resting period during transport was strongly associated with higher load (β = 2.032, 95% CI: 0.921–3.144, *p* < 0.001). Witnessing sick poultry during transport showed a positive but non‑significant association (β = 0.727, 95% CI: −0.210–1.663, *p* = 0.128). Wearing protective clothing was significantly associated with increased load (β = 1.253, 95% CI: 0.184–2.322, *p* = 0.022). The intraclass correlation coefficient was 0.200 (95% CI: 0.050–0.542), indicating that 20% of the variance in *Salmonella* load was attributable to between‑collector differences. Model fit was acceptable (AIC = 324.3). Results are presented in Fig. 3.

**Fig. 3:**
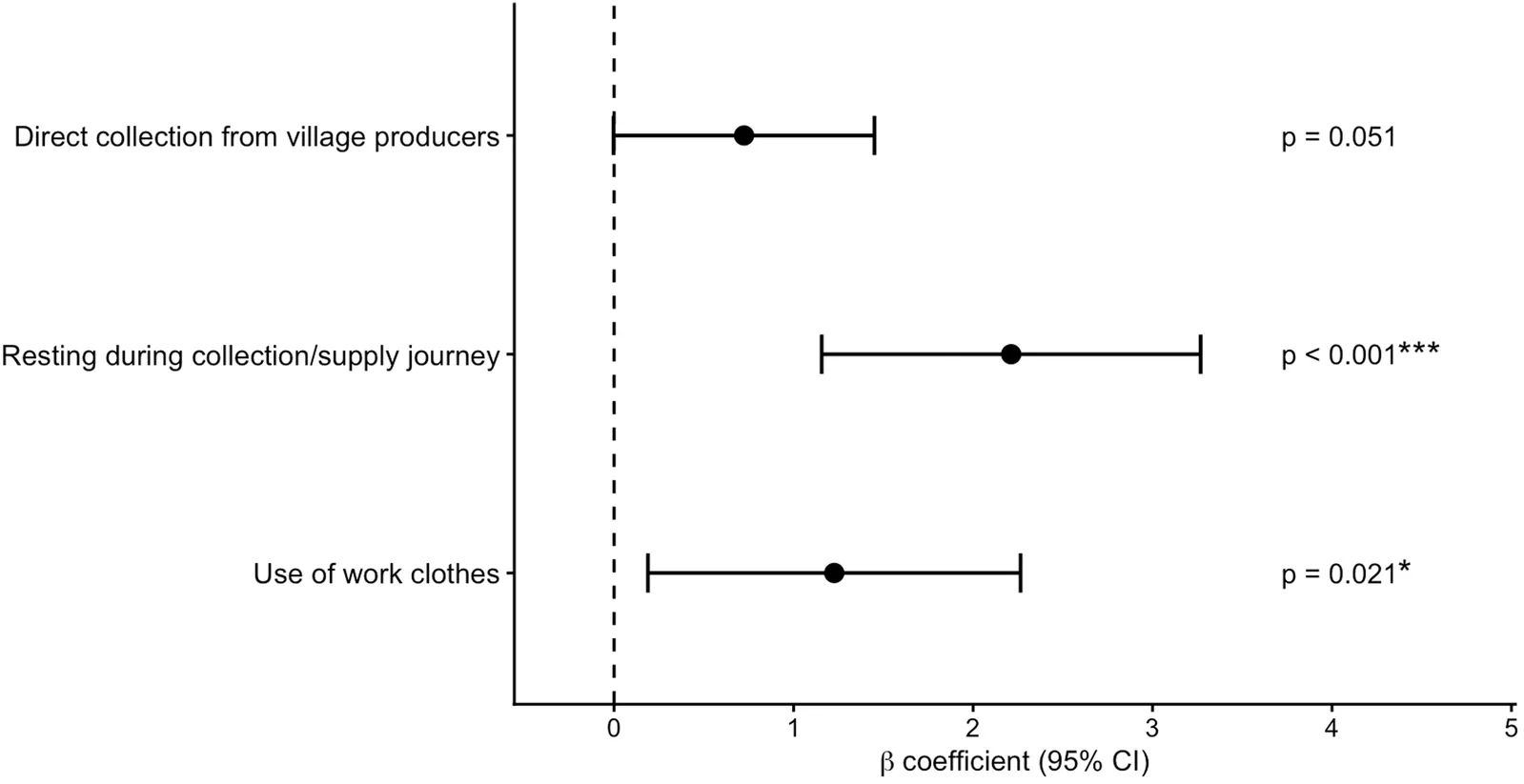
**Forest plot of linear regression coefficients for the quantitative load of *Salmonella* spp. (MPN/g).** Points show estimated β coefficients with 95% confidence intervals. The dashed vertical line indicates the null effect (β = 0). Positive values indicate increased *Salmonella* load, whereas negative values indicate decreased load. Statistically significant associations are indicated by asterisks. (* *p* < 0.05; ** *p* < 0.01; *** *p* < 0.001).

## Discussion

This study found a high occurrence of *Salmonella* spp. among chickens handled by live-bird collectors in Kaya, Burkina Faso. More than two-fifths of sampled chickens and more than half of participating collectors had at least one positive bird. These findings support the hypothesis that the collector stage is an important interface for *Salmonella* spp. Circulation within informal poultry value chains. The study adds to existing evidence by focusing on a relatively neglected group in the poultry value chain. While previous studies have often focused on farms, households, slaughter points, or carcasses, collectors occupy a bridging position between household production systems and urban live-bird markets. Their activities involve aggregation, transport, temporary confinement, handling, and redistribution of birds from multiple sources. These conditions create opportunities for pathogen introduction, mixing, and dissemination.

### *Salmonella* spp. prevalence and associated factors

The animal-level occurrence of Salmonella spp. observed in this study indicates substantial contamination among chickens entering the live-bird market through collector networks. This is consistent with the role of poultry as a reservoir of non-typhoidal Salmonella and with previous studies from informal poultry systems in sub-Saharan Africa.

The overall prevalence of *Salmonella* spp. (41.3%) confirms that poultry collectors represent a key epidemiological node in pathogen dissemination. This estimate is consistent with previous reports from non-formal poultry marketing systems in sub-Saharan Africa, where prevalence ranges between 20% and 60% depending on study design and diagnostic methods (Foley et al., 2011; Ramtahal et al., 2022; Tiendrébéogo et al., 2026). In Burkina Faso, collectors aggregate birds from multiple villages with heterogeneous sanitary conditions, thereby facilitating pathogen mixing and amplification, a dynamic that underscores the relevance of integrated health approaches.

Two factors were independently associated with *Salmonella* detection: reported consideration of vaccination status at purchase and the number of birds transported simultaneously. The association with vaccination status should be interpreted cautiously. In community-based poultry trade in Burkina Faso and across sub-Saharan Africa, vaccination practices are inconsistently applied, poorly documented, and primarily target viral diseases such as Newcastle disease rather than *Salmonella* (Ouedraogo et al., 2026). Thus, this variable likely reflects differences in trading behavior or perceived risk rather than biological protection. Collectors reporting interest in vaccination status may operate within more structured networks or respond to previous disease events, consistent with paradoxical associations described in low‑resource settings (Alali et al., 2010).

The number of birds transported was significantly associated with *Salmonella* spp. positivity, particularly among collectors handling 21-50 birds at a time. This finding is biologically plausible, as higher bird density promotes direct contact, faecal contamination of transport equipment, and stress-induced shedding (Humphrey, 2006; Berghaus et al., 2013). Transport-related stress has been shown to facilitate the shedding and transmission of enteric pathogens in poultry, including *Salmonella* spp. (Van Immerseel et al., 2005). In the context of traditional poultry marketing chains in Burkina Faso, particularly in the Centre-Nord region, transport conditions are often rudimentary, with birds commonly carried in overcrowded cages on motorcycles over long distances, frequently under high ambient temperatures and without adequate ventilation. Field observations further indicated that some collectors especially those operating in the evening accumulate birds at their household compounds overnight before transporting them to markets the following morning. This temporary storage practice may increase mixing between birds from different sources and prolong confinement time, thereby enhancing opportunities for cross-contamination and bacterial shedding.

The absence of a clear dose-response relationship beyond the 21-50 bird category may therefore reflect not only limited statistical power but also heterogeneity in handling practices. In particular, staggered transport strategies and overnight storage may blur the relationship between the number of birds transported at a single time point and the actual cumulative exposure to infection risk.

Although calibration was acceptable, the predictive performance of the logistic regression model remained moderate (AUC = 0.644), suggesting that additional unmeasured factors such as hygiene practices, environmental exposures, or behavioral determinants may contribute to Salmonella occurrence beyond the variables captured in this study.

### *Salmonella* spp. load and associated factors

In contrast to prevalence, factors associated with *Salmonella* load likely reflect mechanisms influencing bacterial amplification among birds that are already infected. In the present study, two variables were significantly associated with increased bacterial load: resting during the collection or supply journey and the reported use of work clothes. In addition, direct collection from village producers showed a borderline positive association with bacterial load.

Resting during the collection or supply journey showed a strong positive association with bacterial load. Although rest periods may be intended to reduce transport stress, their implementation in low-resource trading contexts may inadvertently increase exposure to contaminated environments. Under the field conditions observed in north-central Burkina Faso, resting commonly occurs along roadsides, often under the shade of trees, where birds remain in the same conditions in which they were transported, typically confined in poultry baskets or tied by the legs. As a result, birds remain in prolonged contact with fecal material accumulated in transport containers or on the ground, which may facilitate environmental contamination and bacterial amplification. In addition, resting periods may reflect longer or more complex transport routes, thereby increasing overall confinement time and physiological stress. (Humphrey, 2006; Krüger et al., 2025; Naumovska et al., 2025). Prolonged transport and confinement have been shown to increase the shedding of enteric pathogens such as *Salmonella*, potentially elevating bacterial loads among already infected birds (Berghaus et al., 2013; Maletić et al., 2025). Taken together, these observations suggest that transport and temporary resting practices may represent critical points for bacterial amplification within the live poultry value chain. In informal trading systems where biosecurity measures are limited, prolonged confinement of birds in contaminated transport containers may facilitate the accumulation and dissemination of *Salmonella* spp*.*, thereby increasing the potential for transmission to other birds, traders, and the surrounding environment. However, because resting time may also reflect longer transport duration or more complex collection routes, this variable should be interpreted as capturing broader transport-related exposure rather than the effect of resting alone.

The use of dedicated work clothing was associated with higher *Salmonella* loads. In this study, the variable referred to clothing specifically designated for poultry collection, but without information on whether it was adequately cleaned, disinfected, or replaced. Although washing-related practices were assessed, they were not significantly associated with bacterial load in the present analysis. Therefore, the observed association should not be interpreted as direct evidence that washing frequency itself explained the result. Rather, it may reflect inadequate sanitation not fully captured by the measured variables, or greater occupational exposure among collectors involved in more intensive bird handling and transport activities. In traditional poultry marketing environments, work clothing may be repeatedly reused without proper cleaning and may therefore act as a mechanical vector for pathogen transfer rather than an effective protective barrier. Clothing, footwear, and equipment are recognized as potential fomites contributing to the transmission of *Salmonella* spp. and other enteric pathogens in poultry production and marketing environments (Reta et al., 2023; Feng et al., 2025). Similar observations have been reported in low-resource poultry systems, where the effectiveness of protective measures depends largely on correct use, sanitation practices, and broader biosecurity conditions along the poultry value chain (Thomas et al., 2020; FAO, 2024; Tiendrébéogo et al., 2026).

Overall, these findings highlight how operational practices occurring during poultry collection, transport, and trading can influence not only the occurrence but also the intensity of *Salmonella* spp. contamination in poultry. From an animal-human-environment interface standpoint, such practices may increase the risk of environmental dissemination and onward transmission along informal poultry value chains.

### Comparison with household-level data along the supply chain

The present findings can be further interpreted within a broader poultry value-chain perspective through comparison with a companion study conducted at the household level in the rural commune of Boussouma, Burkina Faso (Tiendrébéogo et al., 2026). Both studies applied identical microbiological procedures (ISO 6579:2012 for detection and MPN for quantification), allowing cautious comparison of contamination patterns across different nodes of the production and marketing chain. Although the sampled populations were not directly linked at the individual bird level and only partially overlapped geographically, this comparison provides useful insights into how *Salmonella* spp. contamination may evolve between production and collection stages.

*Salmonella* spp. prevalence appeared higher at the household level than among birds handled by collectors. Several mechanisms may explain this pattern. Collectors may preferentially purchase birds that appear clinically healthy, thereby excluding visibly diseased animals. In addition, the mixing of birds originating from multiple farms with heterogeneous *Salmonella* positive status may produce a dilution effect at the collector level. Similar dynamics have been described in non-formal poultry trade networks, where trader behavior and flock mixing influence pathogen circulation and apparent prevalence patterns (Grace, 2015; Thomas et al., 2020).

In contrast, the distribution of *Salmonella* loads among positive samples was broadly comparable between the two settings, with similar upper-range bacterial loads observed in both studies. While such extreme values should be interpreted cautiously due to the limited number of positive samples, this observation is consistent with evidence that a subset of birds may carry high bacterial burdens and contribute disproportionately to pathogen transmission. These so-called “super-shedders” have been described as important drivers of *Salmonella* bacterial dynamics in poultry populations (Van Immerseel et al., 2005; Foley et al., 2011).

The determinants of contamination also differed between the two nodes of the value chain. At the household level, risk factors were primarily associated with environmental hygiene and farm management practices, including manure handling, carcass disposal, and water access. In contrast, at the collector level, the factors identified were more closely related to logistics and trading behaviors, such as flock size and bird handling during transport. This divergence supports the concept of node-specific epidemiology, whereby risk factors vary across different stages of the value chain depending on management practices and environmental conditions (Thomas et al., 2020).

From a cross-sectoral health standpoint, these findings highlight the importance of considering the entire poultry value chain when designing control strategies. Interventions targeting a single node are unlikely to sufficiently reduce *Salmonella* circulation, as contamination dynamics are shaped by interactions between farms, collectors, markets, and the surrounding environment. Integrated surveillance and context-adapted interventions across these interconnected nodes are therefore essential to effectively mitigate *Salmonella* risks in informal poultry systems in Burkina Faso.

### Recommendations

The findings suggest several practical entry points for reducing Salmonella spp. circulation at the collector stage of informal poultry value chains. First, cleaning of transport materials should be prioritised. Poultry baskets, crates, ropes, and motorcycle surfaces used to carry birds can accumulate faecal material and may contribute to cross-contamination between batches. Where feasible, cleaning with water and detergent, followed by drying in sunlight, could be promoted as a low-cost intervention. Disinfection may be useful where products are available and used correctly, but recommendations should remain realistic for the local context. Second, prolonged confinement and unnecessary bird mixing should be reduced where possible. Collectors should be encouraged to avoid mixing birds from many different sources in the same container and to minimise the time birds remain tied, crowded, or confined in contaminated transport materials. If temporary stops are unavoidable, birds should not be placed directly on contaminated ground, and transport containers should be kept as clean and ventilated as possible. Third, hygiene messages should include clothing, footwear, and hand hygiene. The finding related to work clothes suggests that personal protective practices need to be accompanied by appropriate cleaning. Dedicated clothing is unlikely to reduce risk if it is repeatedly reused without washing or if it moves between contaminated and clean areas. Simple messages around washing work clothes, cleaning footwear, and handwashing after bird handling could be incorporated into collector training. Fourth, collector-oriented training should be developed in partnership with poultry trader associations, local veterinary services, and market authorities. Training should focus on practical and feasible measures: recognising sick birds, reducing overcrowding, cleaning transport materials, improving temporary holding practices, and reducing contamination during handling and transfer at the market. Finally, future surveillance and intervention studies should include collectors as a distinct value-chain actor. Sampling only at farms, households, slaughter points, or markets may miss important processes that occur during collection and transport.

### Limitations of the study

This study has several limitations. First, the cross-sectional design precludes conclusions about temporality or causality. Associations between transport practices and Salmonella spp. detection or load should therefore be interpreted as exploratory and hypothesis-generating. Second, the study was conducted in one urban commune and during a limited time period. The findings may not be generalisable to other regions, seasons, market systems, or poultry value chains in Burkina Faso. Seasonal variation in poultry movement, temperature, rainfall, and market demand may influence Salmonella spp. circulation and should be assessed in future studies. Third, although four birds were sampled per collector and mixed-effects models were used to account for clustering, the number of collectors was relatively small. This limited statistical power, particularly for variables with sparse categories. The multivariable models should therefore be interpreted cautiously. Fourth, several explanatory variables were based on self-report and may be affected by recall bias or social desirability bias. Some variables, such as vaccination status, were not verified at the individual bird level and may reflect collector perception or purchasing behaviour rather than confirmed biological status. Fifth, the study did not collect detailed individual-level data on bird age, farm of origin, previous antimicrobial exposure, previous disease history, or precise duration of transport. It also did not directly measure microbiological contamination of baskets, crates, ropes, motorcycles, clothing, footwear, or market surfaces. These unmeasured factors may contribute to Salmonella spp. occurrence and load. Finally, although Salmonella spp. was detected and quantified, isolates were not characterised by serotyping or whole-genome sequencing. This limits interpretation of strain diversity, source attribution, antimicrobial resistance profiles, and transmission pathways. Future studies combining bacterial-load estimation with genomic characterisation and environmental sampling would provide deeper insight into Salmonella spp. transmission along informal poultry value chains.

## Conclusions

Chickens handled by live-bird collectors in Kaya frequently carried Salmonella spp., indicating that the collector stage is an important interface for pathogen circulation in informal poultry value chains. By combining detection and bacterial-load estimation, this study provides a more detailed understanding of Salmonella spp. contamination at a neglected point in the poultry supply chain. The findings suggest that Salmonella spp. detection and bacterial load may be associated with different operational practices. Detection was associated with reported consideration of vaccination status at purchase and with the number of birds transported, while higher bacterial load was associated with temporary stops during collection or transport and reported use of work clothes. These findings should be interpreted as exploratory associations rather than causal effects, given the cross-sectional design and reliance on self-reported practices. From a One Health perspective, reducing Salmonella spp. transmission risks in informal poultry systems will require interventions that extend beyond farms and households to include collectors, traders, markets, and the transport environment. Practical collector-oriented measures, including improved cleaning of transport materials, reduced bird mixing, safer temporary holding practices, and hygiene measures for clothing, footwear, and hands, may provide feasible entry points for reducing contamination pressure. Future studies should combine longitudinal sampling, environmental testing, and isolate characterisation to better understand transmission pathways and identify cost-effective interventions for informal live-bird value chains.

## Declarations

### Ethics approval and consent to participate

#### Human participants

Ethical approval for the bird collector survey component of the study was obtained from the ILRI Institutional Research Ethics Committee (IREC; Ref: ILRI-IREC2022-57) and the National Ethics Committee for Health Research of Burkina Faso (Ref: 2022-11-232). The study involving human participants was conducted in accordance with the principles of the Declaration of Helsinki.

For all collector interviews, the project information sheet and consent statement were read and explained to participants in local languages. Informed consent was obtained prior to data collection and recorded electronically using the ODK platform.

#### Animal subjects

Ethical approval for animal handling, sampling, and euthanasia procedures was obtained from the ILRI Institutional Animal Care and Use Committee (IACUC; Ref: ILRI-IACUC2022-38).

All chickens included in the study originated from privately owned village poultry flocks and were sampled while under the custody of bird collectors during collection and transport activities. Access to the birds for sampling was obtained through the participating bird collectors. Participation in the study, including euthanasia of chickens for sampling, was entirely voluntary. Bird collectors were informed in advance about the study objectives and sampling procedures, and their explicit agreement was obtained prior to data and sample collection. Appropriate compensation was provided for each chicken sampled.

Chickens were humanely euthanized by cervical dislocation, performed by trained personnel in accordance with internationally accepted animal welfare guidelines.

#### Availability of data and materials

The data supporting the findings of this research can be obtained from the corresponding author upon reasonable request.

## Funding

This work was funded by the Bureau for Resilience and Food Security of the United States Agency for International Development (USAID) under agreement No. AID-OAA-L-15-00003 as part of the Feed the Future Innovation Lab for Livestock Systems and the Bill & Melinda Gates Foundation, OPP#1175487. Any opinions, findings, conclusions, or recommendations expressed here are those of the authors alone.

## Author contributions

**Wendenso Patrick Bertrand Tiendrébéogo** conceived and designed the study, contributed to data acquisition, analysis and interpretation, and prepared the initial draft of the manuscript. **Assèta Kagambèga, Nicolas Barro**, and **Michel Dione** contributed to the conception and design of the study and critically reviewed the manuscript for important intellectual content. **Arshnee Moodley** contributed to data acquisition and critically reviewed the manuscript for important intellectual content. **Tho Abou Da** and **Rasmané Tao** contributed to data acquisition and reviewed the manuscript. **Brice Ouedraogo** contributed to data analysis and critically reviewed the manuscript for important intellectual content. All authors reviewed and approved the final version submitted for publication and agree to be accountable for all aspects of the work, ensuring that questions related to the accuracy or integrity of any part of the work are appropriately investigated and resolved.

## Acronyms

AIC: Akaike Information Criterion

AVMA: American Veterinary Medical Association

BPW: Buffered Peptone Water

CI: Confidence Interval

ICC: Intraclass Correlation Coefficient

IACUC: Institutional Animal Care and Use Committee

IREC: Institutional Research Ethics Committee

ISO: International Organization for Standardization

MALDI-TOF MS: Matrix-Assisted Laser Desorption/Ionization Time-of-Flight Mass Spectrometry

MPN: Most Probable Number

ODK: Open Data Kit

OR: Odds Ratio

RV: Rappaport-Vassiliadis spp.: species (plural)

## Disclosures

The authors declare that they have no known competing financial interests or personal relationships that could have appeared to influence the work reported in this paper.
